# Analysis of clinical efficacy and safety of hand-sewn anastomosis for the digestive tract with Da Vinci robot in rectal cancer surgery

**DOI:** 10.1186/s12957-023-03172-w

**Published:** 2023-10-10

**Authors:** Zhen Feng, Zhiwei Sun, Qianshi Zhang, Shuangyi Ren

**Affiliations:** Department of Gastrointestinal Surgery, The Second Affiliated Hospital of Dalian Medical University, Dalian, China

**Keywords:** Da Vinci robot, Rectal cancer, Manual anastomosis, Anastomotic leakage, Surgery

## Abstract

**Purpose:**

The study aimed to analyze the clinical efficacy and safety of hand-sewn anastomosis for the digestive tract with Da Vinci robot in rectal cancer surgery.

**Methods:**

A retrospective study was conducted to collect the clinical data from 27 patients who underwent Da Vinci robotic rectal cancer radical surgery in the department of gastrointestinal surgery at the Second Affiliated Hospital of Dalian Medical University from August 2019 to February 2022. All patients received a manual suture for digestive tract reconstruction. After the posterior wall was sutured, the anterior wall was sutured continuously. Finally, a prilling thread was used to sew the junction of the front and rear walls. Perioperative indexes and complications were recorded.

**Results:**

All 27 patients successfully underwent the operation. Neither conversion to laparotomy nor perioperative death occurred. The operation time and intraoperative blood loss were 183.6 ± 44.8 min and 54.8 ± 34.4 ml, respectively. A total of 15.3 ± 7.8 lymph nodes were harvested. The pain score 24 h after operation was 1.3 ± 1.3. The time out of bed, the time to exhaust, and the time to eat were 15.6 ± 2.9 h, 2.2 ± 0.8 days, and 2.1 ± 0.6 days, respectively. A total of 4 patients (14.8%) developed complications after the operation. Grade B anastomotic leakage gradually resolved after drainage and antibiotic therapy in 1 case. A patient with grade C anastomotic leakage received a second operation for ileostomy. One patient with postoperative pneumonia recovered after anti-infective treatment. Another patient with intraperitoneal hemorrhage improved after symptomatic treatment with blood transfusion and hemostasis. The postoperative hospitalization time and total hospitalization costs were 8.9 ± 4.4 days and 89,236.1 ± 13,527.9 yuan, respectively.

**Conclusions:**

Manual suture with Da Vinci robotic surgery system is safe and feasible for reconstructing the digestive tract in rectal cancer surgery.

## Introduction

Da Vinci robotic surgery is one of the latest trends in minimally invasive surgery. It overcomes many technical limitations of traditional laparoscopy and has significant advantages in providing stereoscopic vision, refining surgical operation, and eliminating operation jitter [1]. Since Pigazzi et al. first reported robot-assisted rectal cancer resection in 2006, robot technology has been gradually applied to colorectal cancer surgery [2]. In digestive tract reconstruction during rectal cancer surgery, the application of stapler anastomosis for digestive tract reconstruction is limited due to the short bowel or mesentery for completing an us-preserving surgery for ultra-low rectal cancer. In contrast, the reconstruction of the digestive tract by robot-assisted manual sutures might save more intestinal tissue, and the possibility of anus-preserving surgery for ultra-low rectal cancer is greater. Da Vinci robotic surgery system has the advantages of being less invasive, accurate, and flexible. However, it is very difficult to reconstruct the digestive tract with manual sutures, and high surgical skills of an operator are required. There are few studies on the reconstruction of the digestive tract with manual sutures [3, 4]. We aimed to explore the efficacy of manual suture with Da Vinci robotic system for digestive tract reconstruction in rectal cancer surgery. Therefore, this paper retrospectively analyzed the clinical data of 27 patients who underwent Da Vinci robotic radical rectal cancer surgery and manual anastomosis for digestive tract reconstruction in the Department of Gastrointestinal Surgery at the Second Affiliated Hospital of Dalian Medical University from August 2019 to February 2022.

## Materials and methods

### General profile

There were 261 rectal cancer surgeries in the Department of Gastrointestinal Surgery at the Second Affiliated Hospital of Dalian Medical University from August 2019 to February 2022, including 155 laparoscopic radical rectal cancer surgeries and 106 robotic radical rectal cancer surgeries. Out of 106 patients undergoing robotic radical surgery for rectal cancer, 79 patients underwent instrument anastomosis and 27 patients underwent manual suture. The study was conducted to collect clinical data from 106 patients who underwent Da Vinci robot rectal cancer surgery. Among the 27 patients who underwent hand-sewn anastomosis for the digestive tract with Da Vinci robot in rectal cancer surgery, only 1 patient received preoperative neoadjuvant treatment. There were 13 male and 14 female patients. The average age was 62.11 ± 12.18 years. The average body mass index was 23.4 ± 2.4 kg/m^2^. The average distance from the lower edge of the tumor to the anal margin was 7.2 ± 4.8 cm. The preoperative radiological stages of tumors were as follows: 1 patient had a stage T4 tumor, 10 patients had a stage T3 tumor, 13 patients had a stage T2 tumor, and 3 patients had a stage T1 tumor. Eleven cases had a stage N0 tumor, 6 cases had a stage N1 tumor, and 10 cases had a stage N2 tumor. This study was approved by the Ethics Committee of the Second Affiliated Hospital of Dalian Medical University. Written consent was received from all the patients included.

Inclusion criteria were pathologically confirmed adenocarcinoma, adenoma that cannot be removed by endoscopic submucosal dissection (ESD), neuroendocrine tumor, manual suture for digestive tract reconstruction, and successful anus-preserving surgery. Exclusion criteria comprised cancer combined with other tumors, patients with multiple carcinomas, and familial adenomatous polyposis.

### Operation method

All the patients were operated by the same surgical team. The surgeon in charge has completed 800 Da Vinci robotic gastrointestinal cancer operations. The operation procedure was performed by 3 surgeons. All patients were given general anesthesia with endotracheal intubation. Patients adopted a modified lithotomy position with a low head, high feet, high left arm, and low right arm. The operation followed the principles of total mesorectal excision (TME) and tumor-free operation. The robot used was the third-generation Da Vinci robotic surgery system.

#### Stamp card layout

The five holes method was adopted for the layout of the punch card. In this method, the observation hole (diameter: 12 mm) is located 3–4 cm above the upper right of the umbilical cord. The first arm hole is situated 2–3 cm (diameter: 8 mm) inside the right anterior superior iliac spine at the level of the connection between the two anterior superior iliac spines. The second arm hole (diameter: 8 mm) and the third hole (diameter: 8 mm) are located at the left clavicular midline horizontal to the observation hole and the left anterior axillary horizontal observation hole, respectively. The assistant hole (diameter: 12 mm) is located at the midpoint of the arc connection between the observation hole and the first arm hole. Mechanical arm R1 uses electric scissors, mechanical arm R2 uses a bipolar coagulation gripper, and mechanical arm R3 uses a double-sided perforated gripper. Specific surgical procedures are described below.

#### Lymph node dissection

The retroperitoneum was cut at the right side of the transition between the sigmoid mesocolon and the retroperitoneum at the sacral promontory level. It was freed upward to the root of the inferior mesenteric artery. The inferior mesenteric artery was ligated and severed 1 cm from the root, and its surrounding lymph nodes were cleaned. The inferior mesenteric vein was ligated and severed along the left side of the initial jejunum to the lower edge of the pancreas. The sigmoid colon and descending mesocolon were freed along the left Toldt fusion mesentery space, and the sigmoid mesocolon was cut in a fan-shaped manner to the predetermined resection line. The mesorectum was freed downward: the posterior wall was freed first, and then both sides were freed to the distal 5 cm of the tumor. The mesorectum was freed to the pelvic floor muscle level in low rectal cancer.

#### Reconstruction of the digestive tract

**Fig. 1:**
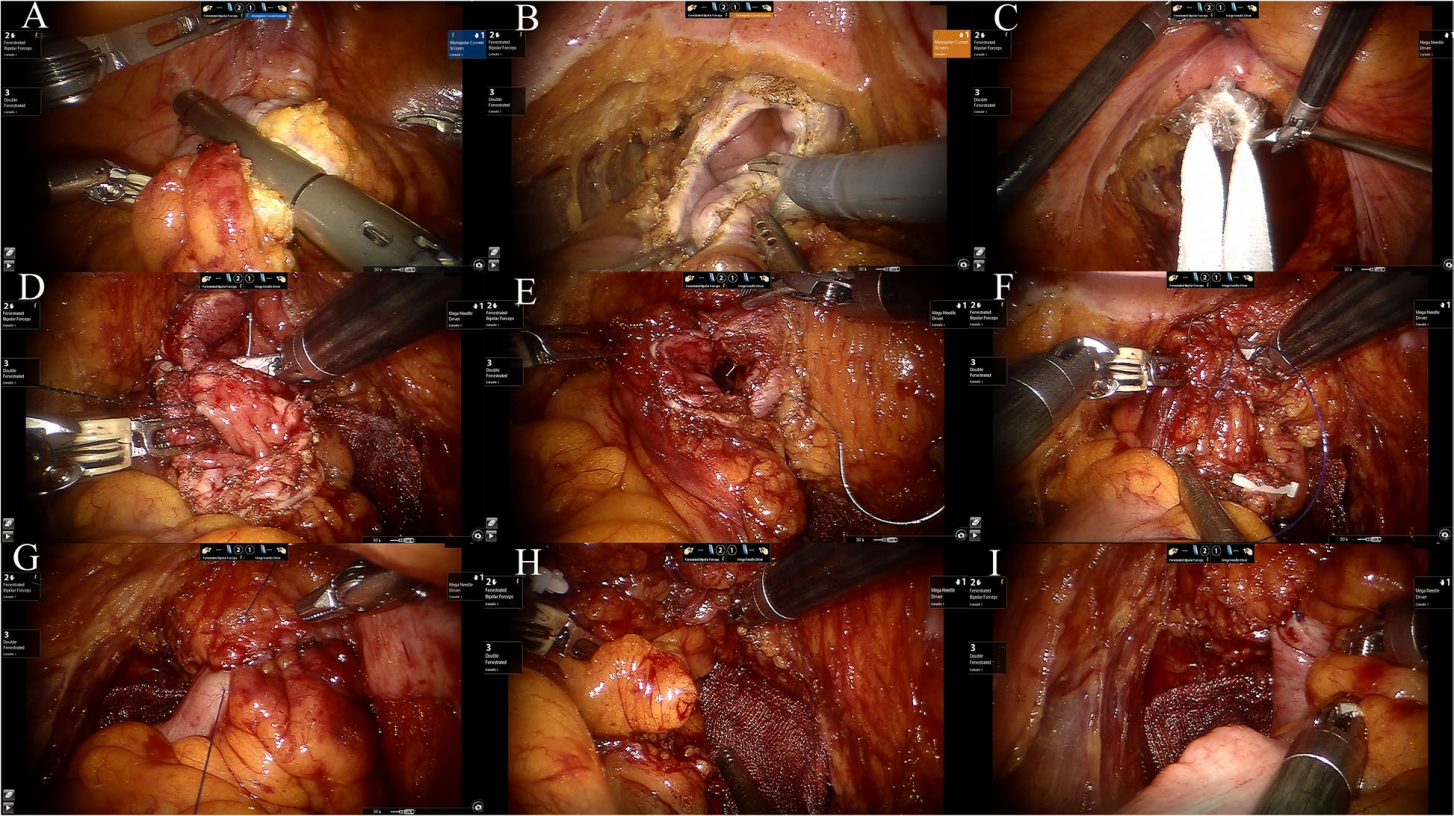
Intracorporeal hand-sewn anastomosis between the sigmoid colon and rectum. The intestinal tube was disconnected and closed with a linear cutting occlude at 10 cm from the proximal end of the tumor (**A**). Electric scissors were used to cut the rectum 2 cm away from the distal end of the tumor (**B**). The specimen was taken out through the anus (**C**). Full-thickness continuous suture of the posterior wall (**D**). Then, the anterior wall was continuously sutured (**E**). Interrupted seromuscular sutures were placed for the junction of the front and rear walls (**F** and** G**). It was checked and ensured that the blood supply of the anastomotic stoma was good and the anastomosis was satisfactory (**H** and **I**)

The rectum was disconnected and closed 2 cm distally to the tumor. Then, a specimen was taken from the abdomen. If the specimen was taken through the natural cavity, the intestinal tube was naked at 10 cm from the proximal end of the tumor. The intestinal tube was disconnected and closed with a linear cutting occluder (Fig. 1A). The blocking clip was used at the distal end of the tumor to block the intestinal canal, and the distal rectum was flushed. Cleaning gauze was put on the back of the rectum, electric scissors were used to cut off the rectum about 2 cm from the distal end of the tumor, and iodophor was used to disinfect the broken end (Fig. 1B). The specimen was taken out through anus or vagina (Fig. 1C). Mechanical arm R1 was replaced with a strong needle holder. The protective sheath of the specimen was placed through the auxiliary hole and prolapsed through the anus. First, the mesorectum was taken out of the body through the specimen protective sleeve. Then, the proximal end of the specimen was clamped, and the specimen was removed through the specimen protective sleeve. The gauze and blocking clip were taken out through the specimen protective sleeve. A clean gauze was placed behind the anastomotic stoma. A new blocking clip was used to block the proximal rectum. The closing nail was cut at the proximal end of the rectum. The posterior wall was sutured continuously from left to right with a 3–0 V-loc suture (Fig. 1D). Then, the anterior wall was sutured continuously with a new V-loc suture (Fig. 1E). The junction of the front and rear walls was sutured with 3–0 prilling thread (Fig. 1F and G). The anastomotic stoma was checked to ensure that the blood supply of the anastomotic stoma was good and the anastomosis was satisfactory (Fig. 1H and I). The blocking clip and closing nail were placed in the specimen bag and taken out through the abdominal puncture hole.

### Observations

Complications included anastomotic leakage, anastomotic bleeding, abdominal bleeding, pelvic infection, intestinal obstruction, and incision infection. Perioperative indicators were operation time, intraoperative blood loss, hospital stay, postoperative hospital stay, time out of bed, exhaust time, eating time, pain score 24 h after operation, and the number of cleaned lymph nodes.

### Analysis of the learning curve

Because there were few patients enrolled in this study, the learning curve analysis used the data of patients undergoing previous Da Vinci robot radical surgery for colorectal cancer. A total of 173 people were used for learning curve analysis, and the learning curve was drawn according to the operation time sequence.

### Statistical methods

The descriptive statistics method was used, and SPSS24.0 statistical software was used for statistical analysis. The measurement data were expressed in mean ± standard deviation, and the counting data were expressed in a constituent ratio (percentage). Excel software and QI Macros 2018 plug-in were used to draw the learning curve.

## Results

### Basic information on the operation

All 27 patients successfully underwent Da Vinci robotic rectal cancer radical surgery with a manual suture for digestive tract reconstruction and no conversion to laparotomy (Table 1). The surgical specimens were taken out through the anus in 14 cases, the vagina in 5 cases, and the auxiliary abdominal incision in 8 cases. Eight patients underwent single-lumen ileostomy. The average operation time was 183.6 ± 44.8 min, and the intraoperative blood loss was 54.8 ± 34.4 ml. A total of 15.3 ± 7.8 lymph nodes were removed.

**Table 1:** Patient’s characteristics and perioperative outcomes (*n* = 27)

Variables	Mean ± SD^a^
Age (years)	62.11 ± 12.18
Sex (male/female)	13/14
Body mass index (kg/m^2^)	23.4 ± 2.4
Distance from the lower edge of the tumor to the anal margin (cm)	7.2 ± 4.8
Operation time (min)	183.6 ± 44.8
Blood loss (ml)	54.8 ± 34.4
Harvested lymph node	15.3 ± 7.8
Postoperative time out of bed (hours)	15.6 ± 2.9
Pain score 24 h after operation	1.3 ± 1.3
Distal margin (cm)	1.7 ± 1.1
Proximal margin (cm)	7.2 ± 3.6
Time of exhaust (days)	2.2 ± 0.8
Time of feeding (days)	2.1 ± 0.6
LOHS^b^ (days)	8.9 ± 4.4
Total hospitalization cost (yuan)	89,236.1 ± 13,527.9

^a^standard deviation

^b^length of hospital stay

### Postoperative recovery

The average time of leaving the bed was 15.6 ± 2.9 h, the pain score was 1.3 ± 1.3 points 24 h after operation, and the time of exhaust and feeding were 2.2 ± 0.8 days and 2.1 ± 0.6 days, respectively. The farthest and nearest incisional margins and circumferential incisional margins of all patients were negative for tumor cells. The farthest and nearest incisional margins were located 7.2 ± 3.6 cm and 1.7 ± 1.1 cm from the incision, respectively. Postoperative pathological staging included 14 stage I patients (51.9%), 3 stage II patients (11.1%), and 7 stage III patients (25.9%). Postoperative pathology showed adenoma in 2 patients and neuroendocrine tumor in 1 patient. The average hospitalization time after operation was 8.9 ± 4.4 days, and the total hospitalization cost was 89,236.1 ± 13,527.9 yuan.

### Postoperative complications

Postoperative complications occurred in 4 patients (14.8%). There were 2 cases of anastomotic leakage. One patient had grade B anastomotic leakage, which gradually resolved after drainage and anti-infection treatment. Another patient, who underwent ileostomy as a second operation, had grade C anastomotic leakage. One case of pneumonia was cured with an anti-infection treatment. There was a patient with intraperitoneal hemorrhage, which improved after symptomatic treatment, such as blood transfusion and hemostasis.

### Comparison between hand-sewn and stapling

There was no statistically significant difference in intraoperative bleeding volume, surgical time, number of lymph nodes cleaned, postoperative exhaust time, incidence of anastomotic leakage, protective ileostomy rate, and hospitalization cost between the two groups of patients. Compared with the instrument anastomosis group, the manual suture group had lower postoperative pain scores at 24 h and earlier postoperative eating time. However, the postoperative hospitalization time of patients in the manual suture group was longer than that in the instrument anastomosis group (Table 2).

**Table 2:** Comparison of perioperative indicators between two groups of patients

Variables	Manual suture group (*N* = 27)	Instrument anastomosis group (*N* = 79)	*P* value
Blood loss (ml)	54.4 ± 34.4	70.4 ± 51.6	0.086
Operation time (min)	183.6 ± 44.8	178.5 ± 42.5	0.421
Harvested lymph node	15.3 ± 7.8	15.8 ± 7.1	0.741
Pain score 24 h after operation	1.3 ± 1.3	3.1 ± 1.3	0.000
Time of exhaust (days)	2.2 ± 0.8	1.9 ± 0.6	0.237
Time of feeding (days)	2.1 ± 0.6	2.6 ± 0.8	0.005
Anastomotic leakage ratio	2/27 (7.4%)	7/79 (8.8%)	1.000
Protective ileostomy rate	8/27 (29.6%)	26/79 (32.9%)	0.815
length of hospital stay after surgery	8.9 ± 4.4	8.0 ± 3.8	0.049
Total hospitalization cost (yuan)	89,236.1 ± 13,527.9	89,567.9 ± 11,361.6	0.574

### Analysis of the learning curve

**Fig. 2:**
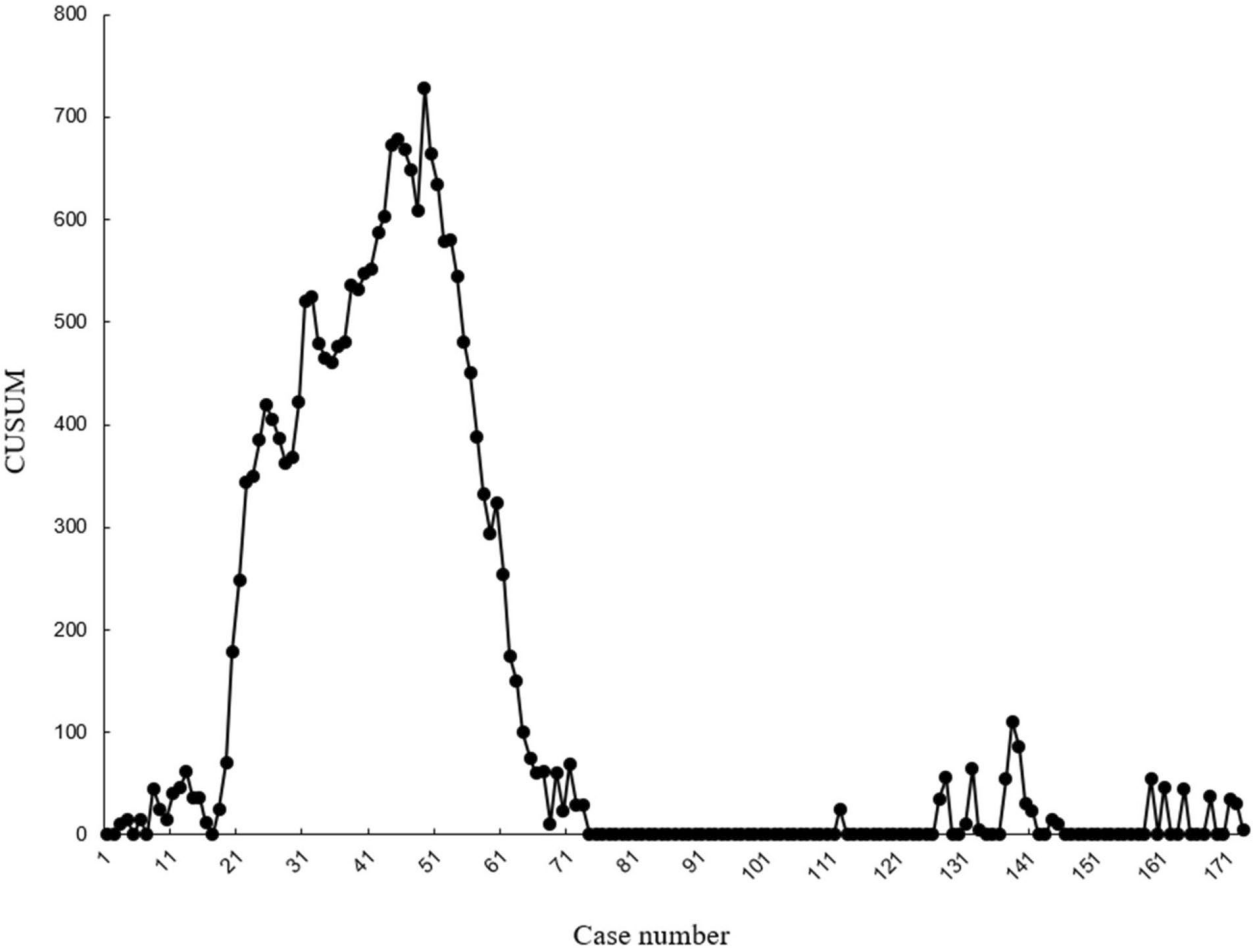
CUSUM analysis for operation time. The case numbers represent the horizontal axis, and the CUSUM value represents the vertical axis

A scatter diagram of the learning curve was drawn. The operation sequence represented the horizontal axis, and the CUSUM value represented the vertical axis. The best fitting equation for the CUSUM learning curve is *y* =  − 0.00804*x*^3^ + 0.42*x*^2^ + 11.8*x* − 101 (*x* represents the case number), and the best value of *R*^2^ is 0.849. The CUSUM curve reaches its peak in the 49th case. Divide the learning curve into the learning period and proficiency period based on this boundary (Fig. 2).

## Discussion

The reconstruction of the digestive tract in the operation of middle and high rectal cancer mainly depends on instrument anastomosis. However, for patients with obesity, pelvic stenosis, and low or even ultra-low rectal cancer, it is extremely difficult to reconstruct the digestive tract with a stapler, and some patients cannot preserve the anus. The reconstruction of the digestive tract by hand suture under a microscope could overcome this problem, but it requires a high level of skill for the operator. Previous studies of the center have shown that complete laparoscopic radical resection of low rectal cancer with manual anastomosis for digestive tract reconstruction is safe and feasible [5]. Compared with laparoscopy, the robotic surgery system has clear and three-dimensional surgical vision, allowing for flexible and stable surgical operation. Besides, it is especially suitable for operations with narrow operating space and high requirements for the anatomical plane. The first multicenter randomized controlled clinical study in China has found that robotic surgery can significantly improve the success rate of anus preservation compared with traditional laparoscopic surgery (82.9% *vs.* 76.8%, *p* = 0.016), which can reduce the positive rate of the circumferential margin, reduce tumor residues, and improve curative effect [6]. Based on these technical advantages, the robotic manual suture should be superior to laparoscopy in digestive tract reconstruction. Recent studies have shown that a laparoscopic manual suture for gastrointestinal reconstruction is safe and feasible in colorectal cancer surgery [3, 5, 7]. However, there is no report on the reconstruction of the digestive tract by manual anastomosis in robotic rectal cancer radical surgery.

Reconstruction of the digestive tract in middle and high rectal cancer is mainly based on instrument anastomosis. However, for digestive tract reconstruction in low rectal cancer, instrument anastomosis is often limited. Among 27 patients in this study, there were 16 cases of low rectal cancer (the distance from the tumor to the anal edge is ≤ 5 cm), 5 cases of middle rectal cancer (the distance from the tumor to the anal edge is > 5 cm but ≤ 10 cm), and 6 cases of high rectal cancer (the distance from the tumor to the anal edge is > 10 cm but ≤ 15 cm). The average distance from the lower edge of the tumor to the anal edge was 7.2 ± 4.8 cm. Radical resection of the tumor requires resection of the distal 2 cm of the intestinal canal of the tumor, and the anal canal is about 2 cm. The instrument anastomosis still needs to waste about 1 cm of the intestinal tube. Hence, the author believes that patients with a distance of ≤ 5 cm from the anal edge should be sutured manually for digestive tract reconstruction.

An increasing number of studies have shown that robotic radical resection of rectal cancer is safe and feasible, with good medium and long-term effects [8, 9]. The results of this study showed that the average operation time of robotic rectal cancer radical surgery was 183.6 ± 44.8 min, the amount of intraoperative bleeding was 54.8 ± 34.4 ml, and the number of cleaned lymph nodes during surgery was 15.3 ± 7.8, which were consistent with the trend matching research results [10, 11]. The ROLARR trial [12] shows that the incidence of anastomotic leakage in robotic rectal cancer surgery is 3.0%. Jing-Jing Li’s study [13] indicates that is 5.4%. The results of this study indicate that the incidence of anastomotic leakage in the manual suture group and the instrument anastomosis group is 7.4% and 8.8%, respectively. There is no statistically significant difference in the incidence of anastomotic leakage between the two groups. However, the incidence of anastomotic leakage in this study is higher than in other studies. The possible reason for the analysis is that this study is a single-center study and the sample size is too small.

Robotic manual suture for digestive tract reconstruction has the following advantages. First, the surgical field is clear, the magnification is larger, and the operator has a 3D vision. Second, the mechanical arm can rotate around 540°, which breaks through the limitation of the movement of both hands and makes the operation more flexible, being especially suitable for surgery in a narrow space. Third, the robotic surgery system can automatically filter out the involuntary tremor of the operator's hand, which makes the operation more stable. Fourth, it can flexibly select the position of the intestinal tube disconnection under direct vision to ensure the negative cutting edge and avoid difficult insertion during the closing period of linear cutting. Fifth, compared with stapler anastomosis, it saves the intestinal canal at the closure nail and anastomosis ring and increases the anus preservation rate of patients with low rectal cancer. Sixth, there is no auxiliary incision in the abdomen. The specimen is taken out through the anus or vagina, and the trauma is smaller. Thus, the patient recovers quickly, and the postoperative hospital stay is short, which conforms to the aesthetic, NOSES, and ERAS concepts. Seventh, the operation cost decreases. Manual suture with 3–0 barb thread for the reconstruction of the digestive tract saves the cost of a cutter and stapler. However, manual suture for the reconstruction of the digestive tract also has some disadvantages. When the manual suture is used for reconstruction, the intestinal tract becomes open and exposed to the abdominal cavity, increasing the chance of abdominal infection. Besides, the lack of tactile sensation of the operator and the tightness or looseness of the suture will affect the healing of the anastomosis. In this study, except for 2 patients with anastomotic leakage, 25 patients did not have an abdominal infection. It indicates that the rectal stump should be protected and fully disinfected during operation, which can effectively avoid the occurrence of abdominal infection. Our remarks regarding surgical experience are as follows. First, before disconnecting the distal intestinal tube, gauze should be placed behind the intestinal tube, and the blocking clip should be used reasonably. The broken end of the intestinal tube should be disinfected with iodophor after the disconnection of the intestinal tube. Second, to disconnect the distal and proximal intestinal tubes of the tumor under the microscope, it is necessary to ensure that the distal resection edge is negative in terms of tumor cell presence to meet the requirements of radical tumor treatment. Additionally, the length of the remaining intestinal tubes should be appropriate. Third, the front and rear intestinal tubes should be of equal length during the reconstruction of the digestive tract. The operator should suture from left to right with 6–8 stitches for each layer, with the needle distance and margin being 0.3–0.5 cm.

To sum up, it is safe and feasible to reconstruct the digestive tract with robotic manual suture in the radical resection of rectal cancer. However, at present, this technology is rarely performed in clinical practice, and its clinical efficacy and prognosis need more evidence-based verification. Manual suture during digestive tract reconstruction by robot surgery requires high technology and can be promoted in some large medical centers.

## Data Availability

Data available on request from the authors: The data that support the findings of this study are available from the corresponding author upon reasonable request.
